# Prognostic Implications of Molecular Subtypes in Primary Small Cell Lung Cancer and Their Correlation With Cancer Immunity

**DOI:** 10.3389/fonc.2022.779276

**Published:** 2022-03-02

**Authors:** Jing Qi, Jiaqi Zhang, Ningbo Liu, Lujun Zhao, Bo Xu

**Affiliations:** ^1^ Department of Biochemistry and Molecular Biology, Tianjin Medical University Cancer Institute and Hospital, Key Laboratory of Cancer Prevention and Therapy, National Clinical Research Center for Cancer, Tianjin’s Clinical Research Center for Cancer, Tianjin, China; ^2^ Department of Radiation Oncology, Tianjin Medical University Cancer Institute and Hospital, Key Laboratory of Cancer Prevention and Therapy, National Clinical Research Center for Cancer, Tianjin’s Clinical Research Center for Cancer, Tianjin, China

**Keywords:** molecular subtype, prognosis, immune microenvironment, immunohistochemistry, small cell lung cancer

## Abstract

**Introduction:**

Small cell lung cancer (SCLC) has recently been characterized as heterogeneous tumors due to consensus nomenclature for distinct molecular subtypes on the basis of differential expression of four transcription markers (ASCL1, NEUROD1, POU2F3, and YAP1). It is necessary to validate molecular subtype classification in primary SCLC tumors by immunohistochemical (IHC) staining and investigate its relevance to survival outcomes.

**Methods:**

Using a large number of surgically resected primary SCLC tumors, we assessed the mRNA and protein levels of the four subtype markers (ASCL1, NEUROD1, POU2F3 and YAP1) in two independent cohorts, respectively. Next, molecular subtypes defined by the four subtype markers was conducted to identify the association with clinicopathologic characteristics, survival outcomes, the expression of classic neuroendocrine markers, and molecules related to tumor immune microenvironment.

**Results:**

Samples were categorized into four subtypes based on the relative expression levels of the four subtype markers, yielding to ASCL1, NEUROD1, POU2F3 and YAP1 subtypes, respectively. The combined neuroendocrine differentiation features were more prevalent in either ASCL1 or NEUROD1 subtypes. Kaplan-Meier analyses found that patients with tumors of the YAP1 subtype and ASCL1 subtype obtained the best and worst prognosis on both mRNA and IHC levels, respectively. Based on multivariate Cox proportional-hazards regression model, molecular subtype classification determined by IHC was identified as an independent indicator for survival outcomes in primary SCLC tumors. Correlation analyses indicated that the four subtype markers in SCLC cancer cells were interacted with its tumor immune microenvironment. Specifically, tumors positive for YAP1 was associated with fewer CTLA4^+^ T cell infiltration, while more immune-inhibitory receptors (FoxP3,PD1, and CTLA4) and fewer immune-promoting receptor (CD8) were found in tumors positive for ASCL1.

**Conclusions:**

We validated the new molecular subtype classification and clinical relevance on both mRNA and protein levels from primary SCLC tumors. The molecular subtypes determined by IHC could be a pre-selected effective biomarker significantly influenced on prognosis in patients with SCLC, which warrants further studies to provide better preventative and therapeutic options for distinct molecular subtypes.

## Introduction

Small cell lung cancer (SCLC) is a particularly aggressive and lethal form of malignancy carcinoma, which predominantly originated from pulmonary epithelial neuroendocrine (NE) cells and characterized by rapid cell division, highly metastatic nature, propensity for acquired therapeutic resistance and exceedingly poor prognosis. While the addition of immunotherapy to standard chemotherapeutics is the first breakthrough in SCLC treatment in over 30 years, the absolute improvements is modest with approximately 2-month in median overall survival, further exploration of the underlying disease mechanisms and refinement of the candidate predictive biomarkers remained to be done (1, 2). Numerous unfavorable results of targeted- and immuno- therapies in SCLC might be due to the insufficient selection of particular patient populations, which in sharp contrast with non-SCLC (NSCLC) that a pre-selected effective biomarker has dramatically altered treatment strategies (3).

SCLC was clinically considered as a single monolithic entity, which was coincide with the emergence of nearly universal deletion or inactivation of TP53 and RB1 at the genetic level (4). Historically, broader analyses of the morphological characteristics, multiple NE phenotype markers and neuronal transcription factors in human SCLC cell lines (5), genetically engineered mouse models (GEMM) (6), and patient-derived xenografts (PDX) (7) were used to interrogate the intratumoral heterogeneity and consequently manifested the potential relevance to consideration of SCLC subgroup stratification. Initial dichotomy between classic SCLC, representing the ASCL1-high subtype, and variant SCLC, representing the NEUROD1-high subtype, was further demonstrated in human tumors and PDX models through clustering of DNA methylation and gene expression profiles (8). Besides these two NE lineage-specific transcription factors, POU2F3, a master transcriptional regulator of tuft cell, was recently identified as a distinct subtype, which expressed exclusively in variant SCLC tumors that lack the expression of classical NE markers (9). An additional molecular subtype consisted of a small unclassified tumors was proposed as driven by YAP1, which is a transcriptional co-activator in the Hippo signaling pathway and preferentially expressed in non-NE cells (10).

Accumulated evidence for these distinct transcriptional factors of SCLC prompts a possibility of therapeutic vulnerabilities towards specific subtype. However, transcriptome-based observations regarding the predicted features of molecular subtypes also revealed the phenomenon of considerable intratumoral subtype overlap (11), which suggested that there is a clonal selection for a dominant transcription factor in the same tumor cell populations. YAP1 was also observed to be positive in stromal cells (12), while bulk RNA sequencing data of primary SCLC tumors failed to identify the specific expression of YAP1 in tumor cells. Notably, immunohistochemical (IHC) methodology provides an objective and visualized metric of molecular subtype markers that allow discriminating the dominant subtype-related markers in tumor cells. Recently, the feasibility of defining the molecular subtype by IHC has been validated in primary SCLC tumors, and the presence of distinct molecular subtype populations also suggested the substantial intratumoral heterogeneity within SCLC tumors, which may potentially assist treatment decisions in SCLC (13). However, the information on previous treatment and survival was missing in their study, hence the need for an independently prognostic inquiry among distinct molecular subtypes. Therefore, we investigated whether molecular subtype markers in primary SCLC tumors may related to the patients’ metastases, survival and the difference in tumor stromal components, immune infiltration or markers of immune activation.

## Materials and Methods

### Patients and Samples Selection

We retrospectively collected 94 frozen tumor samples and 138 formalin-fixed, paraffin-embedded (FFPE) tumor tissue blocks from 232 patients who had consecutively undergone completely surgical resection of primary SCLC in Tianjin Medical University Cancer Institute and Hospital between November 2008 and May 2017. This study has been approved by the ethics committee of Tianjin Medical University Cancer Institute and Hospital with an ethical approval number of bc2021104. All enrolled tumors were pathologically confirmed as SCLC after surgery. Most of 232 tumors were treatment-naive, with only 31 cases underwent chemotherapy at the time of tumor collection. All tumors were pathologically re-staged according to the American Joint Committee on Cancer (AJCC) tumor-node-metastasis (TNM) staging system, 8th edition. One hundred and nine (79.0%) patients subsequently received adjuvant chemotherapy after surgery, with a median cycle number of 4 (range = 1-12 cycles). Characteristics of patients including age, gender, smoking history, preoperative and postoperative treatment history, disease progression during follow-up period were also extracted from the medical records system and telephonic follow-up. The detailed clinicopathologic characteristics of the 94 RNA cohort and 138 FFPE cohort are summarized in **Table 1**.

**Table 1:** Demographic and clinical information related to the tumor samples from two cohorts.

Characteristics	RNA cohort (*N* = 94)	FFPE cohort (*N* = 138)
	No. of patients (%)	No. of patients (%)
Age, years		
≥65	30 (31.9)	37 (26.8)
<65	64 (68.1)	101 (73.2)
Gender		
Female	19 (20.2)	31 (22.5)
Male	75 (79.8)	107 (77.5)
Smoking history		
Smoker	71 (75.5)	114 (82.6)
None-smoker	23 (24.5)	24 (17.4)
Preoperative chemotherapy		
Yes	4 (4.3)	27 (19.6)
No	90 (95.7)	111 (80.4)
Pathological stage		
I-II	77 (81.9)	117 (84.8)
III	17 (18.1)	21 (15.2)

### Real Time Quantitative PCR

Ninety-four RNA samples diagnosed as SCLC by frozen sections were used to subtyping based on their relative expression levels by reverse transcription-quantitative PCR (RT-qPCR). Total RNA was extracted from the frozen tissues using Trizol reagent (Invitrogen) according to the recommended protocol by manufacturer. cDNA was converted using reverse transcriptase from a TUREscript cDNA Synthesis Kit (Aidlab). RT-qPCR was then administered to investigate the gene expression of ASCL1, NEUROD1, POU2F3 and YAP1 using a SYBR-Green assay system. The sequences of the primer sets for real time RT-qPCR were as follows: ASCL1 forward, 5’-TCACCTCTAACACGCACAG-3’ and reverse, 5’-GGCTACTGAGACGAAAGACA-3’; NEUROD1 forward, 5’-GAAAGCCCTCTGACTGATT-3’ and reverse, 5’-GAGAAGTTGCCATTGATGC-3’; POU2F3 forward, 5’-GACCACCATCTCACGATT-3’ and reverse, 5’-GCATCATTCAGCCACTTC-3’; YAP1 forward, 5’-CCTCAGTGTTGTAGCAGTA-3’ and reverse, 5’-GACTCTTAGGTCTCCTTCAG-3’; GAPDH forward, 5’-TGCACCACCAACTGCTTAGC-3’ and reverse, 5’-GGCATGGACTGTGGTCATGAG-3’. The 2−ΔΔCq method was performed to normalize and calculate the relative mRNA amount of target genes to GAPDH.

### Tissue Microarray

For the construction of tissue microarrays (TMAs), the hematoxylin and eosin (HE) whole-slides of 138 cases were reviewed under a light microscope. One representative region contained both dense tumor cells and sufficient mesenchymal cells was selected and circled with highlighters under the microscope, with a diameter of about 3-5mm. The position marked on the HE slides was also marked on the surface of corresponding paraffin block. Then the selected areas on the individual donor paraffin blocks were punched as 2 mm cores to array in 3 recipient paraffin blocks with a biopsy needle. Next, the recipient paraffin blocks were sectioned serially onto “charged” glass slides with a thickness of 4 μm, followed by histocytological reconfirming by HE staining. The remaining microarrays slides were stored at 4°C for subsequent IHC experiment.

### IHC

TMAs sections were first subjected to the oven at 65°C for overnight and then followed by deparaffinized with xylene, rehydrated with an ethanol gradient, and microwave-induced antigen retrieval was performed for 10min at 100°C in citric acid buffer at PH 6.0 or in Tris-EDTA buffer at PH 9.0. Next, endogenous peroxidase activity and nonspecific binding were blocked with 0.3% H_2_O_2_ in methanol for 30 min and 10% goat serum for 30min, respectively. Sections were subsequently treated with 0.1% Triton X-100 for 15min, and rinsed with water followed by incubated with the predetermined, appropriate dilutions of primary antibody overnight at 4°C in a humidified chamber. The detailed primary anti-human protein antibodies with corresponding dilutions used in this study were available in **Supplementary Table 1**. Control tissues were processed in parallel with tissues exposed to the non-immunized serum. The next day, sections were rinsed after rewarming at 37°C for 30min and then incubated with the homologous HRP-conjugated secondary antibody for 30min followed by DAB and hematoxylin staining, respectively. The stained tissue sections were then processed through graded alcohols and xylene, cover-slipped, and allowed to dry at room temperature.

### IHC Scoring Criteria

Except for PD-L1, H-score was adopted to evaluate the immunoreactivities in cases with available results for each marker, which was calculated by multiplying the percentage of stained cells (1-100%) in each intensity category by corresponding intensity of positivity (1 = weak, 2 = moderate, 3 = strong), and the results were summed to yield a range of cumulative score of 0 to 300. Stained slides were observed by three investigators including two pathologists under light microscope using a whole slide of each representative core. The average of H-scores for CD56, Chromogranin A, and Synaptophysin was derived to determine the combined NE score and cases with NE markers unavailable were excluded. Particular attention was paid to the judgment of PD-L1 because 22C3 clone is an approved companion diagnostic antibody in clinic for its predictive role in response to immunotherapy based on a mixture of immune and tumor cell expression (14). The combined positive score (CPS) of PD-L1 is defined by the ratio of the number of all PD-L1-expressing cells (tumor cells, lymphocytes, macrophages) to the number of all tumor cells as the following formula: CPS = (No. PD-L1-stained cells/Total No. viable tumor cells)*100. Tumor cells must exhibit partial or complete membrane staining (≥1+) to be counted as “stained”, whereas immune cells are counted if there is any staining.

### Survival Outcomes

The primary and secondary endpoint of this study were overall survival (OS) and disease-free survival (DFS), respectively. OS was defined as the time from surgery to the date of death or to the time of censored if the patient was still alive. DFS was defined as the time from surgery to the date of the first objective disease progression (local or distant metastasis) or the date of death, whichever occurred first. Outpatient and telephonic follow-up were adopted regularly and the last follow-up time was on September 4, 2020. The median duration of follow-up of all patients was 64.1 months (range = 4.4-117.7 months).

### Statistical Analyses

Unsupervised clustering was performed using package”pheatmap”of R software (version 3.4.4). Venn diagram was drawn using Origin software (version 9.1) to delineate the cross expression among molecular subtype markers. Categorical variables were analysed using chi-square test (χ^2^), χ^2^ correction for continuity or Fisher’s exact test, as appropriate. Survival curves were delineated with the Kaplan-Meier method and differences between curves were estimated by the log-rank test. Variables with P values below 0.1 in the univariate analyses were considered into the multivariate Cox proportional-hazards regression model, with a backward-forward stepwise method. Correlation between continuous variables was calculated using Pearson Correlation Coefficient. All data analyses were conducted with SPSS 24.0 statistical software (IBM Corporation, Armonk, NY, United States) and P-value below 0.05 of the two-sided test was considered significantly different statistically.

## Results

### Expression Level of Four Subtype Markers and Their Prognostic Relevance in RNA Cohort

**Figure 1:**
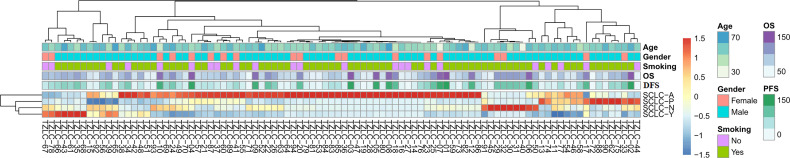
Unsupervised Clustering Analyses of ASCL1, NEUROD1, POU2F3, and YAP1 Gene Expression of Primary Small Cell Lung Cancer Tumors. A total of 94 patients were included to perform this analyses. SCLC-A, SCLC tumors classified into the ASCL1 subtype; SCLC-N, SCLC tumors classified into the NEUROD1 subtype; SCLC-P, SCLC tumors classified into the POU2F3 subtype; SCLC-Y, SCLC tumors classified into the YAP1 subtype. OS, overall survival; DFS, disease-free survival; ASCL1, achaete-scute homologue 1; NEUROD1, neurogenic differentiation factor 1; POU2F3, POU class 2 homeobox 3; YAP1, yes-associated protein 1.

The relative expression levels of ASCL1, NEUROD1, POU2F3, and YAP1 were quantified by RT-qPCR. The mean values (± standard deviation [SD]) of ASCL1, NEUROD1, POU2F3, and YAP1 expression were 2.13 (± 9.68), 2.11 (± 7.95), 0.75 (± 2.22), and 0.23 (± 0.50), respectively. POU2F3 was not expressed in 15 (16.0%) patients and YAP1 was not expressed in 40 (42.6%) patients. Next, unsupervised cluster analyses was performed to identify the dominant marker in SCLC tumors based on the transcriptional levels of the four molecular subtype markers (**Figure 1**). Then, specimens were divided into the following four dominant subtypes: 63 (67.0%) tumors with ASCL1, 10 (10.6%) tumors with NEUROD1, 11 (11.7%) tumors with POU2F3, and 10 (10.6%) tumors with YAP1 subgroup, respectively. Kaplan-Meier analyses identified that patients whose tumors classified into ASCL1 subtype obtained significantly worse OS (P = 0.033) and DFS (P = 0.017) compared to those patients whose tumors with dominant markers of NEUROD1, POU2F3, or YAP1 (**Supplementary Figure 1**), which suggested that molecular subtype classification based on transcriptome data may serve as a predictor for survival outcome in primary SCLC tumors.

### Distribution of Molecular Subtype Markers and Their Correlation With NE Differentiation in FFPE Cohort

**Figure 2:**
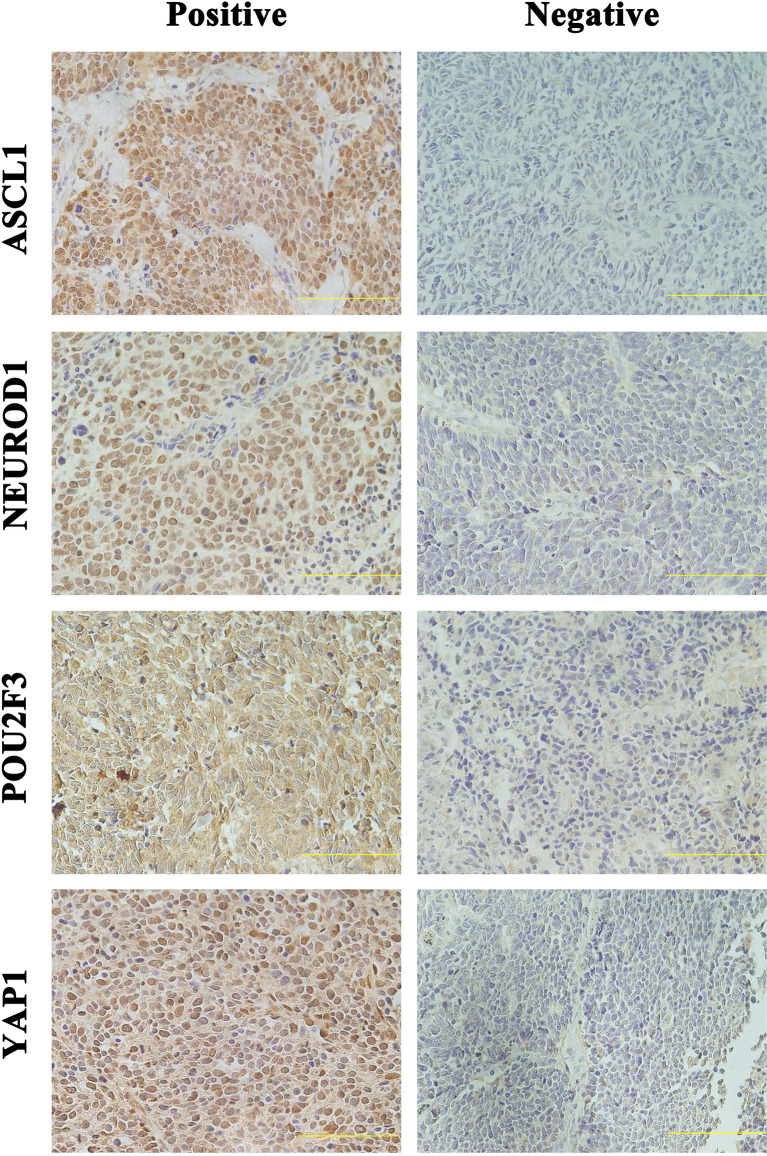
Representative Images of Immunohistochemical Staining in Primary Small Cell Lung Cancer Tumors Positive for One Single Molecular Subtype Marker. Scale bar: 100 μM. Only the tumors with appreciable nuclear staining were considered positive for subtype markers. ASCL1, achaete-scute homologue 1; NEUROD1, neurogenic differentiation factor 1; POU2F3, POU class 2 homeobox 3; YAP1, yes-associated protein 1.

**Figure 3:**
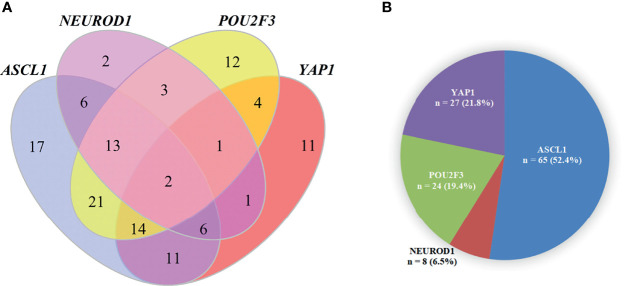
The Pie Chart and Venn Diagram for Subtype Distribution of Primary Small Cell Lung Cancer Tumors. **(A)** A pie chart illustrating the number and percentage of each dominant molecular subtype tumor (total = 124). Fourteen tumors were excluded in this analysis because of detachment during immunohistochemical staining and a dominant marker is defined as the marker with the highest H-score. **(B)** Venn diagram illustrating that most SCLC tumors were positive for multiple molecular subtype markers. ASCL1, achaete-scute homologue 1; NEUROD1, neurogenic differentiation factor 1; POU2F3, POU class 2 homeobox 3; YAP1, yes-associated protein 1.

**Figure 4:**
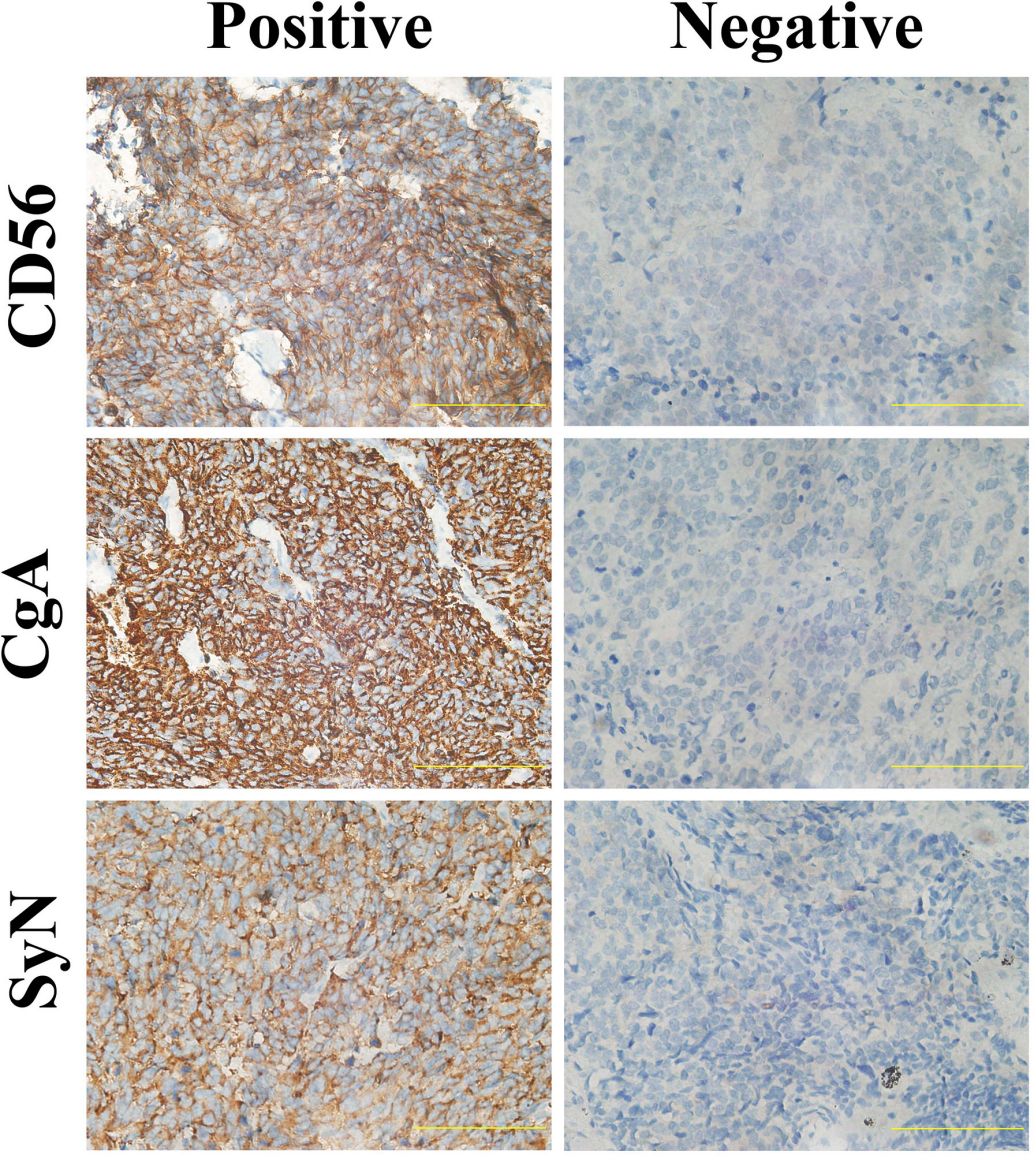
Representative Images of Immunohistochemical Staining for Neuroendocrine Differentiation Markers of Primary Small Cell Lung Cancer Tumors. Scale bar: 100 μM. Cytoplasmic staining of tumor cells was considered positive for SyN and CgA, and tumors with membrane staining were considered positive for CD56. SyN, Synaptophysin; CgA, Chromogranin A.

Detailed results of immunoreactivities for the four molecular subtype markers determined by IHC are described in **Supplementary Table 1**. ASCL1, NEUROD1, POU2F3, and YAP1 was detected to be positive in 93 (74.4%), 34 (27.4%), 71 (56.8%), and 50 (39.7%) of tumors, respectively (**Supplementary Table 2**). ASCL1 and YAP1 exhibited moderate to strong expressions with a mean H-score of 191 (range = 2-294) and 166 (range = 3-285), whereas the H-score of NEUROD1 and POU2F3 showed a relatively weak to moderate expressions with a mean value of 121 (range = 2-270), 145 (range = 2-285). Representative images of IHC staining in primary SCLC tumors for these four markers were shown in **Figure 2**. We observed that most tumors were positive for multiple molecular subtype markers and Venn diagram illustrated the cross expression of these markers in SCLC tumors (**Figure 3A**), which suggested substantial molecular subtype heterogeneity in primary SCLC tumors. ASCL1 exhibited coordinated expression with the other three molecules in 81.1% of ASCL1-positive cases. POU2F3 showed a higher co-expression rate (55.6%) than NEUROD1 (30.0%) and YAP1 (36.7%) in ASCL1-positive cases. A total of 124 samples with available H-scores were used to compare the relative expression levels of the four molecular subtype markers, and a dominant marker is defined as the marker with the highest H-score. A breakdown of molecular subtype composition revealed that ASCL1 is the dominant subtype in 52.4% of the tumors, followed by YAP1 (21.8%), POU2F3 (19.4%), and NEUROD1 (6.5%) (**Figure 3B**). Furthermore, we found that the average combined NE score of tumors in either POU2F3 (median = 88) or YAP1 (median = 63) subtypes were dramatically lower than tumor in either ASCL1 (median = 165) or NEUROD1 (median = 156) subtypes, which was consistent with previous report (12, 15) and suggested that the degree of NE differentiation may contribute to the evolution of molecular subtypes. Representative images of IHC staining for NE differentiation markers are shown in **Figure 4**.

### Relationship of Molecular Subtypes to Clinicopathologic Features and Survival Outcome in FFPE Cohort

To investigate the relationship between molecular subtypes and clinicopathologic factors, we compared patients’ characteristics in the SCLC tumors grouped by dominant subtype markers in FFPE cohort (**Table 2**). We did not observed any significant correlation between molecular subtypes and age, gender, smoking history, or pathological stage (all P > 0.05), and there were no statistical differences in preoperative chemotherapy, postoperative treatment strategy, and postoperative chemotherapy cycle among SCLC subtypes (all P > 0.05). However, we found that SCLC molecular subtypes were closely related with the risk of brain metastasis (P = 0.035), liver metastasis (P = 0.033), and bone metastasis (P = 0.033), respectively. Specifically, more patients whose tumors of the ASCL1 subtype had significantly increased risks of either brain (P = 0.011) or bone (P = 0.037) metastasis than those of the YAP1 subtype. Pairwise comparison analyses also found that the incidence of liver metastasis (P = 0.048) in tumors of the NEUROD1 subtype was more prevalent compared to tumors of the YAP1 subtype.

**Table 2:** Comparison of baseline clinicopathologic characteristics among molecular subtypes of primary SCLC tumors in FFPE cohort.

Characteristics	ASCL1-subtype	NEUROD1-subtype	POU2F3-subtype	YAP1-subtype	*P* value
	No. of patients (%)	No. of patients (%)	No. of patients (%)	No. of patients (%)	
Age, years					0.453
≥65	15 (23.1)	3 (37.5)	9 (37.5)	6 (22.2)	
<65	50 (76.9)	5 (62.5)	15 (62.5)	21 (77.8)	
Gender					0.394
Female	17 (26.2)	1 (12.5)	5 (20.8)	3 (11.1)	
Male	48 (73.8)	7 (87.5)	19 (79.2)	24 (88.9)	
Smoking history					0.533
Smoker	51 (78.5)	6 (75.0)	21 (87.5)	24 (88.9)	
None-smoker	14 (21.5)	2 (25.0)	3 (12.5)	3 (11.1)	
Preoperative ChT					0.907
Yes	13 (20.0)	1 (12.5)	4 (19.0)	4 (14.8)	
No	52 (80.0)	7 (87.5)	20 (81.0)	23 (85.2)	
Pathological stage					0.590
I-II	53 (82.8)	8 (100)	20 (83.3)	22 (81.5)	
III	11 (17.2)	0 (0)	4 (16.7)	5 (18.5)	
Treatment after surgery					0.814
None	10 (16.9)	0 (0)	1 (4.3)	1 (4.3)	
ChT	26 (44.1)	4 (57.1)	12 (52.2)	14 (60.9)	
ChT+TRT	16 (27.1)	2 (28.6)	7 (30.4)	5 (21.7)	
Others	7 (11.9)	1 (14.3)	3 (13.0)	3 (13.0)	
No. of adjuvant ChT					0.321
≥4	34 (58.6)	5 (71.4)	16 (80.0)	17 (70.8)	
<4	24 (41.4)	2 (28.6)	4 (20.0)	7 (29.2)	
Brain metastasis					0.035
No	35 (68.6)	6 (85.7)	16 (88.9)	22 (95.7)	
Yes	16 (31.4)	1 (14.3)	2 (11.1)	1 (4.3)	
Lung metastasis					0.122
No	42 (84.0)	6 (85.7)	16 (94.1)	23 (100)	
Yes	8 (16.0)	1 (14.3)	1 (5.9)	0 (0)	
Liver metastasis					0.033
No	44 (86.3)	5 (71.4)	17 (100)	23 (100)	
Yes	7 (13.7)	2 (28.6)	0 (0)	0 (0)	
Bone metastasis					0.033
No	39 (78.0)	6 (85.7)	16 (94.1)	23 (100)	
Yes	11 (22.0)	1 (14.3)	1 (5.9)	0 (0)	
Adrenal metastasis					0.121
No	47 (94.0)	5 (71.4)	17 (100)	21 (91.3)	
Yes	3 (6.0)	2 (28.6)	0 (0)	2 (8.7)	

ChT, chemotherapy; SCLC, small cell lung cancer; TRT, thoracic radiotherapy treatment; ASCL1, achaete-scute homologue 1; NEUROD1, neurogenic differentiation factor 1; POU2F3, POU class 2 homeobox 3; YAP1, yes-associated protein 1.

**Figure 5:**
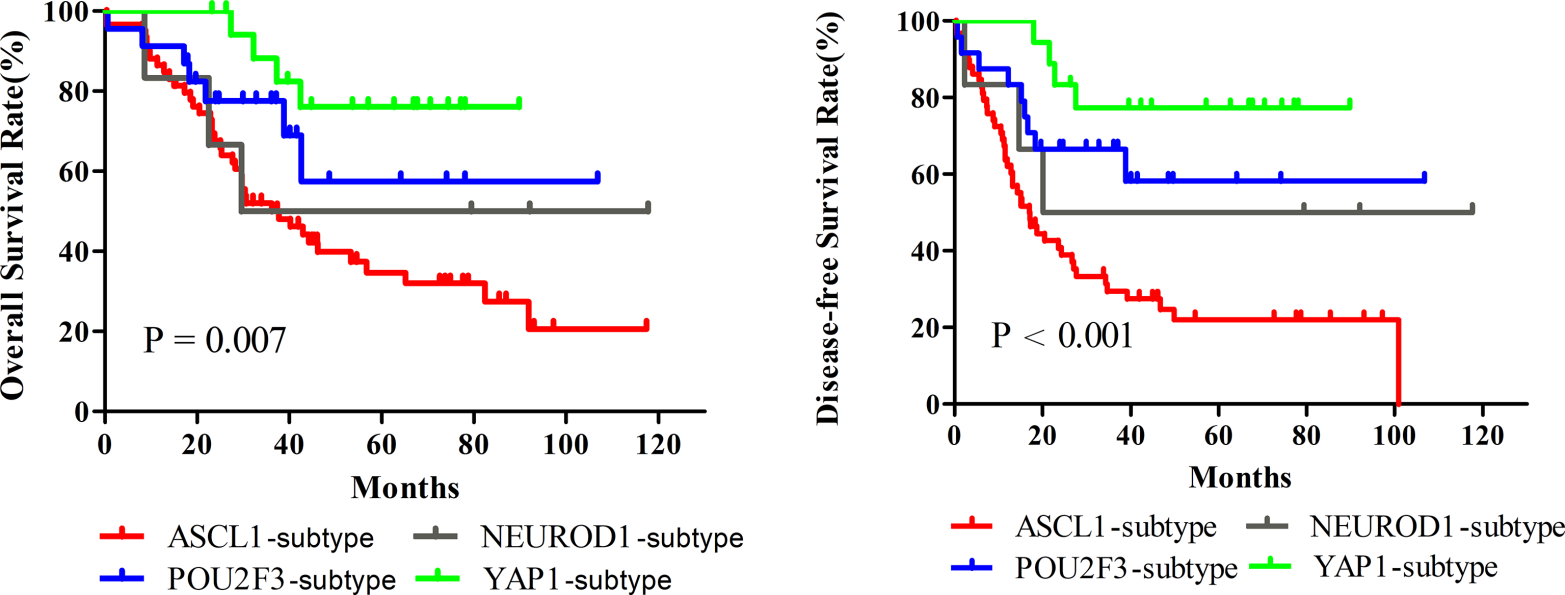
Survival Comparison among Dominant Molecular Subtypes determined by Immunohistochemical Staining of Primary Small Cell Lung Cancer. A total of 124 patients were utilized to perform Kaplan-Meier analyses, and fourteen tumors were excluded in this analysis because of detachment during immunohistochemical staining. ASCL1, achaete-scute homologue 1; NEUROD1, neurogenic differentiation factor 1; POU2F3, POU class 2 homeobox 3; YAP1, yes-associated protein 1.

Next, we investigated whether SCLC molecular subtypes determined by IHC might have potential impacts on patients’ survival. What stood out was that the OS (P = 0.007) and DFS (P < 0.001) in patients classified into the YAP1 subtype significantly prolonged compared to the remaining subtypes. **Figure 5** and **Table 3** detailed the specific survival time of patients with distinct subtypes. A relatively better survival was observed in patients whose tumor of the POU2F3 subtype. The corresponding 3-year OS was 82.4% for YAP1subtype and 68.9% for POU2F3 subtype. Inferior prognosis were obtained in patients classified into NEUROD1 and ASCL1 subtypes, with comparable 3-year OS of about 48.9% and 50.1%, respectively. In addition, Kaplan-Meier analyses revealed that the lower level of combined NE score indicated a better OS than did the higher group (P = 0.085). Collectively, these results reveal that molecular subtype is a predictive indicator in human primary SCLC and suggested that molecular subtype is associated with degree of NE differentiation.

**Table 3:** Survival comparison of molecular subtypes determined by immunohistochemical staining in primary small cell lung cancer tumors.

Survival Outcome	ASCL1-subtype	NEUROD1-subtype	POU2F3-subtype	YAP1-subtype	*P* value
Overall survival					0.007
Median(months)	37.7	29.5	—	—	
1-year(%)	86.4	80.3	89.3	—	
3-year(%)	50.1	—	68.9	82.4	
5-year(%)	34.7	—	—	—	
Disease-free survival					0.000
Median(months)	16.9	20.0	—	—	
1-year(%)	61.1	67.8	83.3	95.7	
2-year(%)	38.3	—	—	80.6	
3-year(%)	28.0	—	61.3	—	

ASCL1, achaete-scute homologue 1; NEUROD1, neurogenic differentiation factor 1; POU2F3, POU class 2 homeobox 3; YAP1, yes-associated protein 1.

### Univariate and Multivariate Analyses: Combining Clinicopathologic Variables and Molecular Subtypes

To investigate whether the prognostic correlation of SCLC molecular subtypes was affected by other clinicopathologic factors in this cohort, univariate and multivariate Cox’s regression analyses were performed and the results were summarized in **Table 4**. Univariate analyses showed that metastasis occurred in brain (HR = 3.243, P = 0.000), lung (HR = 2.453, P = 0.024), liver (HR = 7.566, P = 0.000), bone (HR = 2.365, P = 0.015), and adrenal (HR = 3.626, P = 0.009) were predictors of inferior OS. Patients received adjuvant chemotherapy less than 4 cycles (HR = 0.340, P = 0.000) also had a significantly worse OS. In addition, the survival analyses for DFS as functions of the smoking history (HR = 2.258, P = 0.043), brain metastasis (HR = 4.503, P = 0.000), lung metastasis (HR = 3.661, P = 0.000), liver metastasis (HR = 4.108, P = 0.000), bone metastasis (HR = 4.206, P = 0.000), and adjuvant chemotherapy less than 4 cycles (HR = 0.515, P = 0.015) showed a significant correlation with disease progression.

**Table 4:** Univariate and multivariate analyses for prognostic significance of clinical features and molecular subtypes in patients with primary small cell lung cancer.

Variables	OS				DFS			
	Univariate analyses	*P*	Multivariate analyses	*P*	Univariate analyses	*P*	Multivariate analyses	*P*
	HR (95% CI)		HR (95% CI)		HR (95% CI)		HR (95% CI)	
Clinical markers								
Age, ≥65 *v.* <65 years	1.284 (0.720-2.290)	0.397			1.152 (0.672-1.978)	0.606		
Gender, female *v.* male	0.533 (0.295-1.095)	0.087	0.233 (0.052-1.051)	0.058	0.583 (0.302-1.127)	0.109		
Smoking, smoker *v.* non-smoker	2.069 (1.929-4.606)	0.053	4.349 (0.960-19.691)	0.056	2.258 (1.027-4.966)	0.043	2.881 (1.039-7.986)	0.042
Preoperative ChT, yes *v.* no	1.118 (0.522-2.394)	0.775			0.911 (0.474-1.751)	0.780		
Pathological stage, I-II *v.* III	0.774 (0.400-1.501)	0.449			0.953 (0.534-1.702)	0.872		
No. of adjuvant ChT, ≥4 *v.* <4	0.340 (0.190-0.609)	0.000			0.515 (0.302-0.879)	0.015		
Brain metastasis, yes *v.* no	3.243 (1.710-6.148)	0.000			4.503 (2.515-8.064)	0.000	2.711 (1.290-5.701)	0.009
Lung metastasis, yes *v.* no	2.453 (1.125-5.348)	0.024			3.661 (1.863-7.195)	0.000	2.610 (1.185-5.748)	0.017
Liver metastasis, yes *v.* no	7.566 (3.139-18.235)	0.000	2.975 (1.036-8.541)	0.043	4.108 (1.903-8.866)	0.000		
Bone metastasis, yes *v.* no	2.365 (1.181-4.738)	0.015	2.445 (0.977-6.118)	0.056	4.206 (2.289-7.728)	0.000		
Adrenal metastasis, yes *v.* no	3.626 (1.371-9.589)	0.009			2.301 (0.971-5.455)	0.058		
Molecular subtypes	0.652 (0.498-0.855)	0.007	0.599 (0.420-0.853)	0.005	0.601 (0.468-0.773)	0.000	0.640 (0.461-0.888)	0.008

ChT, chemotherapy; OS, overall survival; DFS, disease-free survival; HR, hazard ratio; CI, confidence interval; ASCL1, achaete-scute homologue 1; NEUROD1, neurogenic differentiation factor 1; POU2F3, POU class 2 homeobox 3; YAP1, yes-associated protein 1.

Next, clinicopathologic variables with P value less than 0.1 in univariate analyses as well as molecular subtypes were included in a multivariate Cox proportional-hazards regression model. Multivariate analyses revealed that molecular subtype classification was an independent prognostic factor significantly influenced on OS (HR = 0.599, P = 0.005) and DFS (HR = 0.640, P = 0.008), respectively. We also found several clinical factors, such as smoking history, brain metastasis, lung metastasis, and liver metastasis, were strongly correlated with inferior survival outcome. Together, these results reveal that the molecular subtype classification determined by IHC could be a reliable biomarker for stratifying SCLC patients into different survival outcome.

### The Expression Correlation of Immunologic Indicators in Tumors With Single Subtype Markers of Primary SCLC

**Figure 6:**
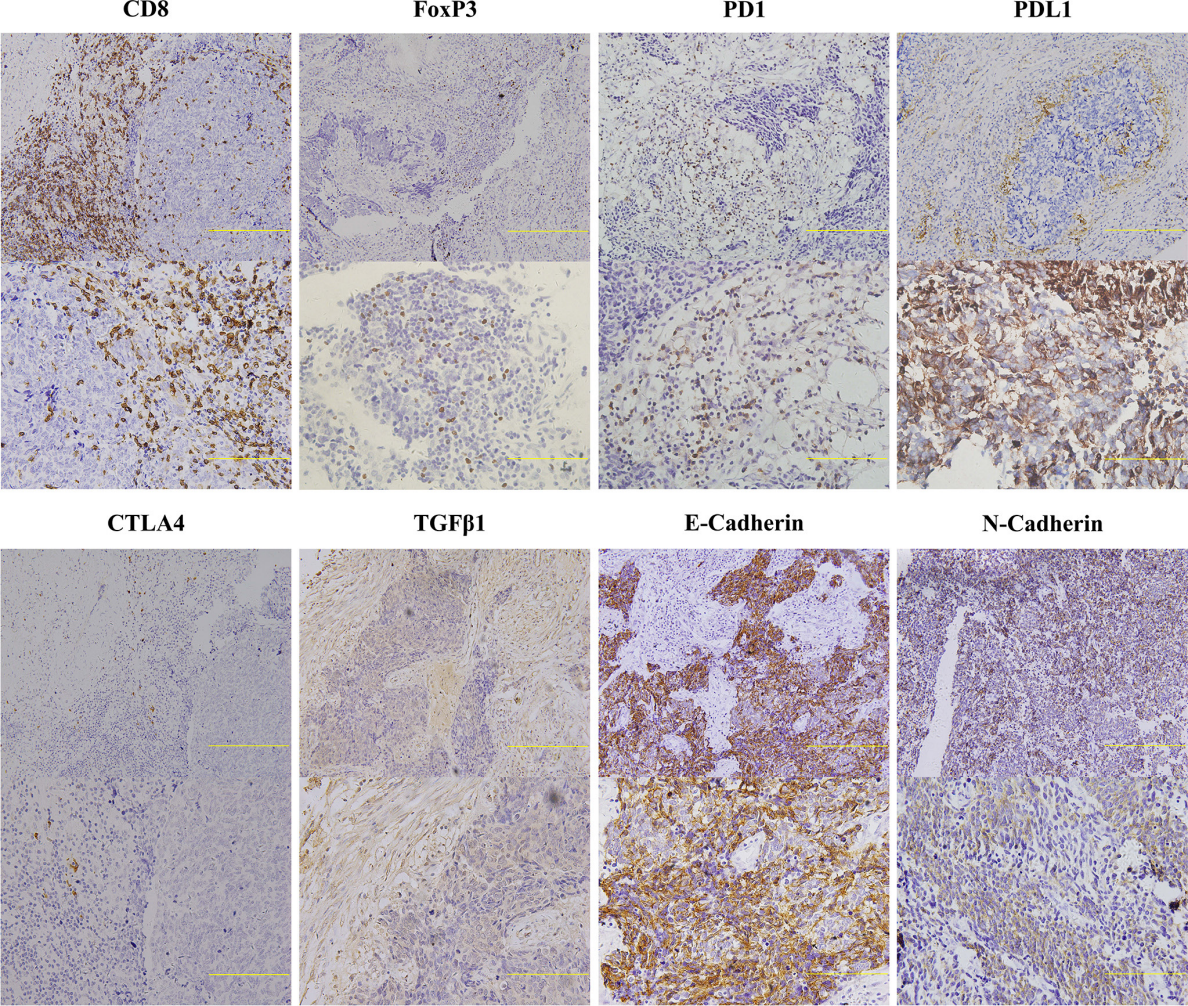
Representative Images of Immunohistochemical Staining for Indicators Related to Cancer Immunity of Primary Small Cell Lung Cancer Tumors. Two immunohistochemical staining images with different scanning resolutions were selected for each immune-related marker (upper image: 20×, lower image: 40×). Scale bar: 100 μM. Only the lymphocytes with appreciable membrane staining were considered positive for CD8, PD1, PDL1, and CTLA4, and samples positive for FoxP3 were defined as appreciable nuclear staining observed in lymphocytes. Tumors with membrane staining were considered positive for E-Cadherin, N-Cadherin, and TGFβ1. Extracellular staining for TGFβ1 was also considered to be positive. FoxP3, forkhead box P3; CTLA4, cytotoxic T lymphocyte antigen 4; PD1, programmed cell death 1; PDL1, programmed cell death ligand 1; TGFβ1, transforming growth factor beta 1.

In order to explore the associations of tumor immune microenvironment in tumors with single subtype markers, we performed IHC for immune-inhibitory receptors (FoxP3, PD1, PDL1, and CTLA4) as well as immune-promoting receptor (CD8), and then quantified their staining results according to corresponding evaluation methods. We also assessed the expression levels of three proteins (E-Cadherin, N-Cadherin, TGFβ1) associated with tumor progression by IHC score. Representative images of IHC staining in primary SCLC tumors positive for these proteins are shown in **Figure 6**. Using their corresponding original H-scores, we compared the correlations between these proteins and available single subtype markers by Pearson correlation coefficient (**Table 5**). The results revealed that ASCL1 expression levels had a significantly correlation with infiltrating levels of CD8^+^ T cells (r = -0.229, P = 0.011), FoxP3^+^ Treg cells (r = 0.380, P = 0.000), PD1^+^ T cells (r = -0.255, P = 0.005) and CTLA4^+^ T cells (r = 0.260, P = 0.005). In SCLC tumors with distinct subtype markers, the correlations were not all the same. NEUROD1 expression levels had a strongly correlation with infiltrating levels of PDL1^+^ tumor (r = -0.200, P = 0.035). POU2F3 expression levels positively correlated with infiltrating levels of CTLA4^+^ T cells (r = 0.302, P = 0.001). YAP1 expression levels positively correlated with the levels of CTLA4^+^ T cells (r = -0.200, P = 0.036). The IHC scores of either ASCL1 or NEUROD1 were strongly and positively associated with the levels of TGFβ1, respectively, which has been reported to prevent immune cells from entering tumors by inducing fibrosis (16). We did not observed obvious associations of these four subtype markers with indicators involved in epithelial-mesenchymal transition (E-Cadherin and N-Cadherin). Collectively, these results suggested that the protein levels of these four subtype markers in SCLC cancer cells are interacted with its tumor immune microenvironment.

**Table 5:** The expression correlation of immunologic indicators in tumors positive for single subtype marker of primary small cell lung cancer.

Variable	ASCL1		NEUROD1		POU2F3		YAP1	
	*r*	*P*	*r*	*P*	*r*	*P*	*r*	*P*
CD8	-0.229	0.011	0.074	0.424	0.045	0.619	-0.101	0.271
FoxP3	0.380	0.000	0.171	0.067	0.130	0.161	0.077	0.413
PD1	-0.255	0.005	-0.085	0.358	-0.104	0.257	-0.160	0.086
PDL1	-0.062	0.514	-0.200	0.035	0.080	0.401	-0.068	0.478
CTLA4	0.260	0.005	0.093	0.329	0.302	0.001	-0.200	0.036
E-Cadherin	0.093	0.306	-0.131	0.152	0.172	0.056	0.053	0.565
N-Cadherin	0.127	0.160	0.071	0.439	0.068	0.450	0.137	0.134
TGFβ1	0.237	0.011	0.208	0.028	0.056	0.511	0.174	0.058

ASCL1, achaete-scute homologue 1; NEUROD1, neurogenic differentiation factor 1; POU2F3, POU class 2 homeobox 3; YAP1, yes-associated protein 1; FoxP3, forkhead box P3; PD1, programmed cell death 1; PDL1, programmed cell death ligand 1; CTLA4, cytotoxic T lymphocyte antigen 4; TGFβ1, transforming growth factor beta 1.

## Discussion

A classification of SCLC tumors based on differential expression of transcription regulators for molecular subtypes has emerged as an evolving area of investigation, which probably may paving the way for potentially personalized therapeutic approaches for this deadly form of lung cancer. Whether SCLC molecular subtypes are associated with different metastatic organ-tropisms and distinct patients outcomes, as well as the complex interplay between each transcription factors and immune infiltration remained unclear. In the present study, we ascertained the differential expression of ASCL1, NEUROD1, POU2F3, YAP1 on a large number of surgically resected SCLC tumors both on RNA and protein levels and then performed an evaluation of the prognostic value between dominant subtype markers, followed by an examination on differential expression in several cell surface proteins, including CD8, FoxP3, PDL1, CTLA4, etc.

We observed comparable distributions of ASCL1 and NEUROD1 subtypes both on mRNA and protein level, respectively. The slight disagreement in distributions of POU2F3 and YAP1 subtypes might originated from a possibilities of a clonal selection for a dominant transcription factor during tumor expression (17), as well as the inclusion of more combined SCLC histology samples in FFPE cohort, which was reported containing more POU2F3 and YAP1 expression specimens (12). As reported by Taofeek (18), we confirmed that molecular subtypes determined by IHC were an independent indicator for survival outcome in primary SCLC patients, and the best survival benefit of SCLC tumors had an YAP1 subtype, followed by POU2F3 and ASCL1 subtypes, which exhibited consistent tendency in mRNA and protein levels. Meanwhile, brain and bone metastasis were less prevalent in patients classified into YAP1 subtype than those with ASCL1 subtype, respectively, and liver metastasis was decreased in YAP1 subtype than NEUROD1 subtype. The prognosis of NEUROD1 subtype on mRNA and protein level remained inconsonant and fuzzy due to a minor subset of tissues. In particular, the usefulness of NE differentiation between molecular subtypes as an indicators was evaluated and suggested that tumors with POU2F3 and YAP1 subtypes were relevant to lower combined NE phenotype, whereas ASCL1 and NEUROD1 subtypes were relevant to higher combined NE phenotype, suggesting the possibility of at least two different cells of origin. We also observed that the combined NE score tended to be higher in the group with a worse prognosis, which is in accordance with the observation of the study by Hamanaka (19). Consistently, we observed that CTLA4, represented inhibited immune regulators, was negatively correlated with YAP1 expression, while positively correlated with POU2F3 and ASCL1 expression, respectively. Besides, several proteins, including CD8^+^ T cells, FoxP3^+^ T cells and PD1^+^ T cells, exhibited a significant correlation with ASCL1 expression, which suggested that there is a theoretically possibility for a administration of combined immunotherapy based on ASCL1 expression, as supported by a evidence of immunosuppression.

The non-NE sutype, featured by YAP1 and POU2F3 expression, has contributed to the heterogeneity of SCLC tumors by accumulated evidence. YAP1 was disclosed to be associated with decreased drug sensitivity in cell lines (10, 20) and POU2F3 expression represented a distinct profile subset of SCLC arising from or recapitulating the differentiation of the tuft cells (9). In addition, YAP1 subtype cell lines are consistently mesenchymal, while the POU2F -subtype cell lines have a epithelial signature (20). Sun et al. reported that increased YAP1 expressions, were indicators of prolonged survival in lung cancer and esophageal cancer, whereas were related to a poorer prognosis in gastric cancer and pancreatic cancer (21). Taofeek et al. revealed that, compared with the other three subtypes, YAP1 subtype of SCLC harbored a better outcome for OS and PFS, which may account for the enrichment of high interferon-γ and T-cell-inflamed gene expression profile (18). However, insignificantly statistical difference for OS or DFS were observed between the four subtypes due to minor cases in their study. YAP1 subtype also acquired the longest survival period in this research and YAP1 expression determined by IHC was negatively associated with the expression of CTLA4, an important molecule in inhibiting T cell activity. Recently, a discovery by McColl used wildtype RB1 mutation status as a surrogate marker of YAP1 expression and revealed that wildtype RB1 was associated with significantly shorter OS and PFS compared to patients with mutant RB1, in which with an obvious decreased chemo-refractory SCLC tumors (10). Since RB1 status was not detected in our study, we were curious to scrutiny this apparently discrepancy of their study and found that nearly half of RB1 expression did not co-express YAP1 both in cell lines and tumors, which may explain the reverse trend towards survival of YAP1 expression.

Recently, a few studies has been investigated the distribution pattern and prognostic value of ASCL1 expression in SCLC tumors at the protein level by IHC, yielding to a broader range of 42.5-80% for ASCL1 expression and a significant tendency of prolonged OS for ASCL1 negative group (22–24). Herein, the destructive effect of ASCL1 subtype on OS and DFS were also obtained both in mRNA and protein levels. Additionally, binary group comparisons indicated that the risk of distant metastasis in ASCL1 subtype was 2.262 times higher than non-ASCL1 subtype. The tumor-promoting effect of ASCL1 expression in our study was consistent with previous observations in lung adenocarcinoma, in which ASCL1-positive is accompanied with a poor immune cell infiltration based on both transcriptomic and IHC analyses, thereby mediates its cell-proliferation effect and primarily resistant to immunotherapy (25, 26). This pattern of immunosuppressive phenomenon were also observed and were significantly correlated with elevated levels of FoxP3^+^ T cells, CTLA4^+^ T cells, PD1^+^ T cells, accompanied with reduced level of CD8^+^ T cells, which suggested that there is a theoretically possibility for inferior prognosis of this subtype.

As an additional phenomenon, the H-scores of ASCL1 and NEUROD1 showed positively association with the level of TGFβ1 in SCLC specimens. TGFβ1, an isoform of TGFβ, has been reported to have bi-directional roles in cancer progression (27) as well as exert suppressive and pleiotropic effects on the immune system, including the regulation of T cells, natural killer cells, and macrophages (28). It has been reported that TGFβ-mediated apoptosis was suppressed due to the epigenetic silencing of TGFβ type II receptor (TβRII) by high level of EZH2, leading to an up-regulation of ASCL1 in a Smad-dependent manner followed by an accelerated proliferation of SCLC cells *in vivo* and vitro (29), which is in accordance with the inferior prognosis of ASCL1 positive patients and the significant correlation between ASCL1 and TGFβ1 in our analyses. However, the expression of EZH2, TβRII and Smad were not detected concurrently. Stimulation by the addition of NEUROD1 to the primary cultured pancreatic acini also facilitates endocrine trans-differentiation towards insulin positive cells along with the increasing expression of EGR and TGFβ (30). Further exploration of the role and mechanisms of TGFβ in response to the ASCL1 and NEUROD1 activation are needed in the future.

This study not only an evaluation on the prognostic role in molecular subtypes of SCLC in two independent cohorts, but also a description of the discrepant immune microenvironment among single subtype markers at the protein level, based on a substantial numbers of surgically resected SCLC specimens. Nevertheless, there are still some limitations in this retrospective study, including the common disadvantage of incompletely tumor tissue in TMAs, the different selection of antibodies, the evaluation criteria of protein expression and the unavoidable bias of various treatment regimens. An integrated analyses of prognostic association between molecular subtypes and tumor immune microenvironment on genetic level is warranted in the future to provide more comprehensive information of SCLC. In conclusion, this reproducible categorization and consensus nomenclature begun to shed light on our in-depth understanding of the molecular features and distinct prognosis of SCLC, which may provides insights into screening differential drug sensitivities, building a reliable and valuable molecular subtype drivers to assign SCLC patients to each of the clinical trials and eventually determine their unalloyed prognostic and therapeutic value.

## Conclusion

Molecular subtypes determined by IHC could be an powerful, economical, and practical biomarker for independently stratifying SCLC patients into differential prognosis.

## Data Availability Statement

The raw data supporting the conclusions of this article will be made available by the authors, without undue reservation.

## Ethics Statement

The human participants involved in this study have been reviewed and approved by the ethics committee of Tianjin Medical University Cancer Institute and Hospital with an ethical approval number of bc2021104. Written informed consent for participation was not required for this study in accordance with the national legislation and the institutional requirements.

## Author Contributions

JQ and JZ have contributed equally to this work. JQ, JZ, and LZ designed the study. JQ, JZ, NL, and BX participated in data collection, data analyses, software, and interpretation. JQ, JZ, NL, LZ, and BX wrote this manuscript or made critical revisions to the pivotal intellectual content. All authors have approved the submission of the final manuscript.

## Conflict of Interest

The authors declare that the research was conducted in the absence of any commercial or financial relationships that could be construed as a potential conflict of interest.

## Publisher’s Note

All claims expressed in this article are solely those of the authors and do not necessarily represent those of their affiliated organizations, or those of the publisher, the editors and the reviewers. Any product that may be evaluated in this article, or claim that may be made by its manufacturer, is not guaranteed or endorsed by the publisher.
