# Supplementary material for: Prognostic Implications of Molecular Subtypes in Primary Small Cell Lung Cancer and Their Correlation With Cancer Immunity
Source: Front Oncol. 2022 Mar 2;12:779276. doi: 10.3389/fonc.2022.779276 (PMC8924463; doi:10.3389/fonc.2022.779276)
Supplement: Supplementary file 1 [file DataSheet_1.doc]

Supplementary Table 1: The Detailed Primary Anti-human Protein Antibodies with Corresponding Dilutions and Scoring Criteria Used in immunohistochemical staining

| Antibody | Product code | Manufacturer | Dilution | Scoring criteria |
| --- | --- | --- | --- | --- |
| ASCL1 | Ab211327 | Abcam | 1:100 | H-score of nuclear labeling was scored as positive reactivity. |
| NEUROD1 | Ab213725 | Abcam | 1:1000 | H-score of nuclear labeling was scored as positive reactivity. |
| POU2F3 | Ab191840 | Abcam | 5x | H-score of nuclear labeling was scored as positive reactivity. |
| YAP1 | Ab52771 | Abcam | 1:50 | H-score of nuclear and cytoplasmic labeling were scored as positive reactivity. |
| SyN | ZA-0506 | ZSGB-BIO | 1:200 | H-score of cytoplasmic labeling was scored as positive reactivity. |
| CgA | ZM-0076 | ZSGB-BIO | 1:150 | H-score of cytoplasmic granular labeling was scored as positive reactivity. |
| CD56 | ZM-0057 | ZSGB-BIO | 1:200 | H-score of membranous labeling was scored as positive reactivity. |
| CD3 | Ab135372 | Abcam | 1:150 | H-score of membranous labeling was scored as positive reactivity. |
| CD8 | Ab93278 | Abcam | 1:200 | H-score of membranous labeling was scored as positive reactivity. |
| FoxP3 | 320201 | Biolegend | 1:50 | H-score of nuclear labeling was scored as positive reactivity. |
| CTLA4 | Ab227709 | Abcam | 1:100 | H-score of membranous labeling was scored as positive reactivity. |
| PD1 | Ab52587 | Abcam | 1:50 | H-score of membranous labeling was scored as positive reactivity. |
| PDL1 | 22C3 | Agilent Dako | 1:50 | CPS was calculated as described in the Methods. |
| TGFβ1 | Ab92486 | Abcam | 400x | H-score of membranous and extracellular labeling were scored as positive reactivity. |
| E-Cadherin | #14472 | Cell Signaling | 1:100 | H-score of membranous labeling was scored as positive reactivity. |
| N-Cadherin | #13116 | Cell Signaling | 1:125 | H-score of membranous labeling was scored as positive reactivity. |

ASCL1, achaete-scute homologue 1; NEUROD1, neurogenic differentiation factor 1; POU2F3, POU class 2 homeobox 3; YAP1, yes-associated protein 1. SyN, Synaptophysin; CgA, Chromogranin A; FoxP3, forkhead box P3; CTLA4, cytotoxic T lymphocyte antigen 4; PD1, programmed cell death 1; PDL1, programmed cell death ligand 1; TGFβ1, transforming growth factor beta 1.


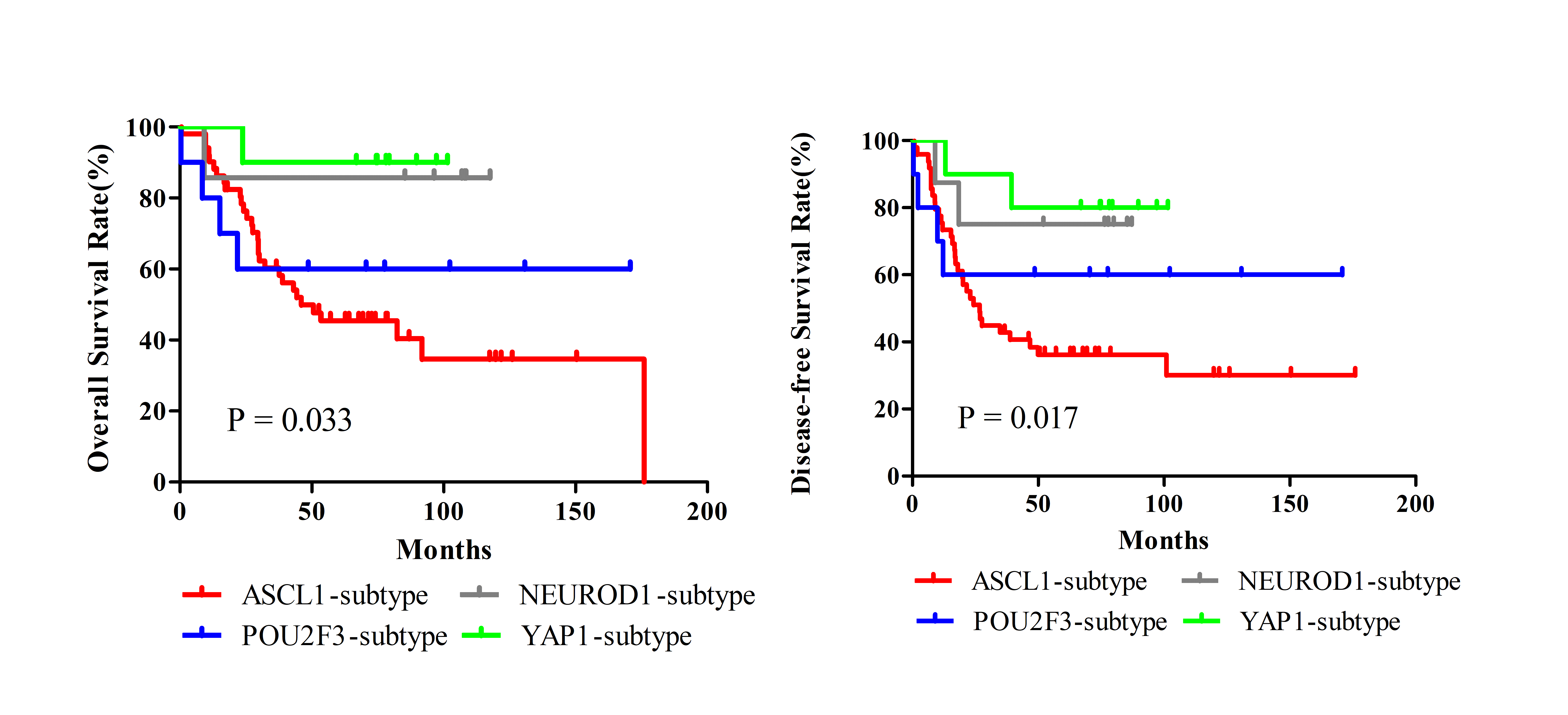


Supplementary Figure 1: Kaplan-Meier Curves of Overall Survival, Disease-Free Survival between Molecular Subtypes of Small Cell Lung Cancer in RNA Cohort. A total of 94 patients were included to perform this analyses. OS, overall survival; DFS, disease-free survival; ASCL1, achaete-scute homologue 1; NEUROD1, neurogenic differentiation factor 1; POU2F3, POU class 2 homeobox 3; YAP1, yes-associated protein 1.

Supplementary Table 2: Protein Expression Profiles of Tumors Positive for the Four Subtype Markers in Small Cell Lung Cancer

| Marker | Total number of tumors | Positive cases, N(%) | H-score range, N(% positive cases) | | | Mean H-score(Full Range) |
| --- | --- | --- | --- | --- | --- | --- |
|  |  |  | 1-100 | >100-200 | >200 |  |
| ASCL1 | 125 | 93 (74.4) | 14 (15.1) | 34 (36.6) | 45 (48.4) | 191 (2-294) |
| NEUROD1 | 124 | 34 (27.4) | 18 (52.9) | 11 (32.4) | 5 (14.7) | 121 (2-270) |
| POU2F3 | 125 | 71 (56.8) | 18 (25.4) | 45 (63.4) | 8 (11.3) | 145 (2-285) |
| YAP1 | 126 | 50 (39.7) | 12 (24.0) | 26 (52.0) | 12 (24.0) | 166 (3-295) |

ASCL1, achaete-scute homologue 1; NEUROD1, neurogenic differentiation factor 1; POU2F3, POU class 2 homeobox 3; YAP1, yes-associated protein 1.
